# Expression Profiling Reveals Genes Involved in the Regulation of Wool Follicle Bulb Regression and Regeneration in Sheep

**DOI:** 10.3390/ijms16059152

**Published:** 2015-04-23

**Authors:** Guangbin Liu, Ruize Liu, Xiaohui Tang, Jianhua Cao, Shuhong Zhao, Mei Yu

**Affiliations:** 1 Key Lab of Agricultural Animal Genetics, Breeding and Reproduction of Ministry of Education, Huazhong Agricultural University, Wuhan 430070, China; E-Mails: gbliu@scau.edu.cn (G.L.); lrz_2003@163.com (R.L.); jhcao@mail.hzau.edu.cn (J.C.); shzhao@mail.hzau.edu.cn (S.Z.); 2 College of Animal Science, South China Agricultural University, Guangzhou 510642, China; 3 College of Animal Science, Agriculture and Animal Husbandry College of Tibet, Linzhi, Tibet 860000, China; E-Mail: xzlztxh2011@163.com

**Keywords:** hair follicle, wool, sheep, gene expression profiling, high-throughput RNA sequencing

## Abstract

Wool is an important material in textile manufacturing. In order to investigate the intrinsic factors that regulate wool follicle cycling and wool fiber properties, Illumina sequencing was performed on wool follicle bulb samples from the middle anagen, catagen and late telogen/early anagen phases. In total, 13,898 genes were identified. KRTs and KRTAPs are the most highly expressed gene families in wool follicle bulb. In addition, 438 and 203 genes were identified to be differentially expressed in wool follicle bulb samples from the middle anagen phase compared to the catagen phase and the samples from the catagen phase compared to the late telogen/early anagen phase, respectively. Finally, our data revealed that two groups of genes presenting distinct expression patterns during the phase transformation may have important roles for wool follicle bulb regression and regeneration. In conclusion, our results demonstrated the gene expression patterns in the wool follicle bulb and add new data towards an understanding of the mechanisms involved in wool fiber growth in sheep.

## 1. Introduction

Hair (including wool, fleece and alpaca) is an important material in textile manufacturing, and it is produced from the hair follicle (HF), which is invaginated in skin [1]. The structure of the hair follicle is complex [1,2,3,4]. Basically, each hair follicle can be divided into two parts: one is the upper permanent region, and the other is the lower regenerating region, including the hair follicle bulb containing the hair matrix cells (HMCs), which give rise to the hair. Modulated by extra-follicular macroenvironmental factors, the hair follicle bulb undergoes growth cycles, including anagen (growth), catagen (regression) and telogen (rest) phases. In the anagen phase, the proliferating signals from the dermal papilla cells (DPCs) induce the HMCs to proliferate and differentiate into the hair shaft (HS) and the inner root sheath (IRS). At the end of anagen, the speed of HMCs proliferation and differentiation declines, and then, the cells at the lower regenerating region of the hair follicle enter an apoptosis phase, termed catagen. At this stage, the hair follicle becomes recessionary, and the hair follicle bulb condenses and moves upward. Cells, including HMCs, undergo the apoptosis process. The telogen phase follows the catagen phase. In telogen, the proliferation and differentiation of most of cells stop, and the hair follicle stays in quiescence. By the end of telogen, the hair follicle bulb starts to regenerate, and the hair follicle remodels back into its growth phase state (anagen), restarting the next growth cycle. Therefore, given that hair is produced from the rapidly proliferating hair follicle bulb, it is of importance to identify the intra-follicle bulb expressed genes that function to enhance or suppress the growth process of the hair follicle bulb and hair fiber.

Due to the economic importance of wool as hair fiber in textile manufacturing, many studies have identified the genes associated with the growth and properties of wool fiber in sheep and goat, such as *DSG1* [5], *ILK* [6], *BMP4*, *FGF10* [7], *GH-R*, *IGF-1*, *IGF-IR* [8], *KRTs* and *KRTAPs* [9,10]. These genes could be used to increase the production and/or alter the properties of the wool fiber, therefore improving the economic efficiency of wool production. Recently, the genes and pathways of *Wnt*, *Shh*, *TGF-*β, *BMP* and *Notch*, which are mainly secreted by the skin and adipose tissue, have been shown to be involved in hair follicle development and cycling of growth [11]. Several reports on goat and sheep skin transcriptome analyses identified that some genes and pathways may be important for the regulation of anagen phase reentry of the hair cycle and coat or skin color [12]. In addition, the DPCs that are surrounded by the hair follicle have been shown to play an important role in the regulation of hair follicle regeneration via the Wnt signaling pathway [13]. Research based on the skin transcriptome data aimed at elucidation of the mechanisms underlying curly fleece formation in Tan sheep suggested related candidate genes, including several keratin gene family members [14]. These research studies yielded some insights into the regulation factors derived from the extra-follicular macroenvironment in goat and sheep. Those factors modulate the periodical proliferation and differentiation of cells in the hair follicle bulb to make new hair shafts and inner root sheaths. However, the intrinsic activators/inhibitors within the hair follicle bulb that regulate HF cycling and hair fiber properties in response to the extra-follicular macroenvironmental signaling remain to be understood.

The present study was conducted to investigate the gene expression patterns in the sheep hair follicle bulb, which gives rise to the hair fiber, during the cyclical phases of middle anagen, catagen and late telogen/early anagen. Our analyses revealed that two groups of genes with distinct expression patterns might be involved in the apoptosis regulation in the catagen phase and keratinocyte differentiation in the anagen phase. These results provide information for the genes that might be important for wool follicle bulb regression and regeneration, as well as wool fiber growth during the wool follicle cycling in sheep.

## 2. Results and Discussion

### 2.1. Results

#### 2.1.1. Illumina Sequencing and qPCR Validation

**Figure 1:**
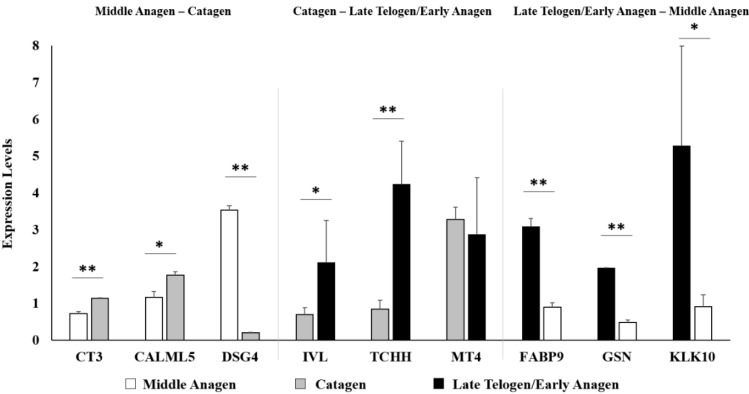
Validation of the sequencing results by qPCR. Nine differentially expressed genes with RPKM (reads per kilobase of exon per million fragments mapped) >5 were selected for validation of the sequencing results by quantitative real-time PCR (qPCR). The results showed good agreement between qPCR and sequencing data (** *p* < 0.01, * *p* < 0.05).

**Figure 2:**
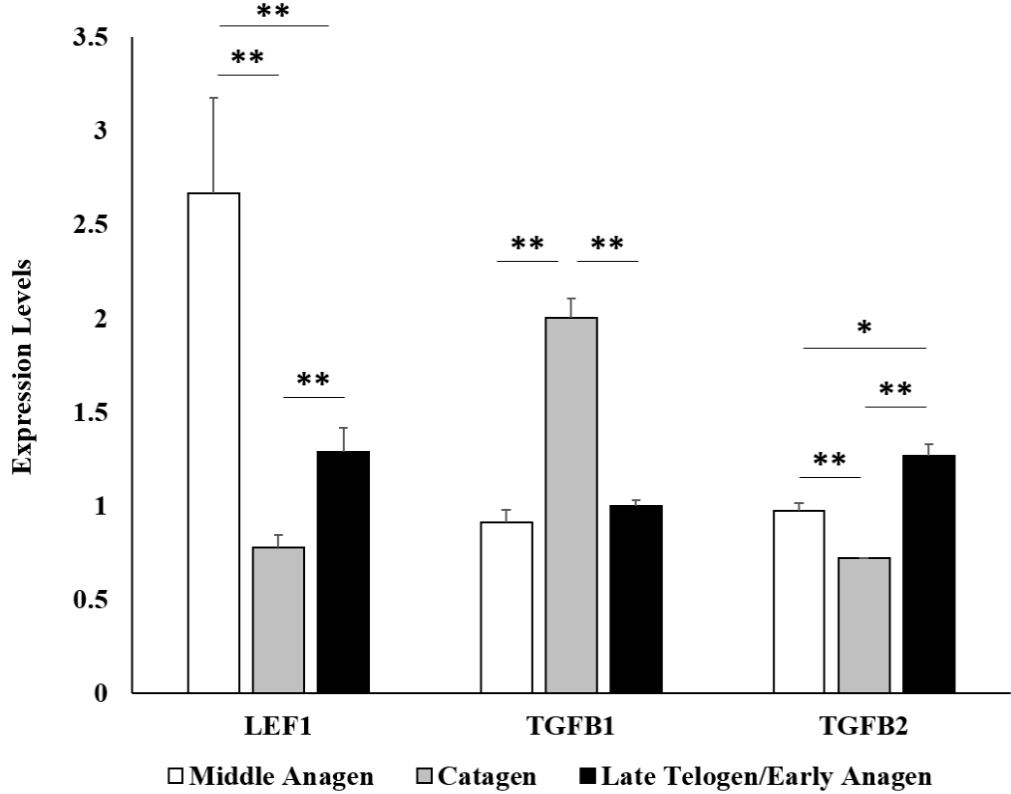
Validation of the expression levels of *LEF1*, *TGFB1* and *TGFB2* marker genes by qPCR. The expression levels of *LEF1*, *TGFB1* and *TGFB2* genes were checked by qPCR, to confirm that our samples were collected from the right phases. Previous studies showed that the expression level of *LEF1* in hair follicles is higher in anagen compared with the catagen and telogen phases [15], whereas the expression level of *TGFB1* is upregulated in catagen [16], and the expression level of *TGFB2* is upregulated in late telogen/early anagen [17]. Both the qPCR results and sequencing data showed that the expression patterns of these three genes were consistent with the expected phases (** *p* < 0.01,* *p* < 0.05).

Wool follicle bulb samples of Tibetan sheep from the middle anagen (*n* = 3), catagen (*n* = 2) and late telogen/early anagen (*n* = 2) phases, respectively, were used for Illumina sequencing. About 10–15 million sequencing clean reads (quality-filtered and the adapters removed) per sample were generated. After annotation and normalization of the read count, a total of 13,898 genes were obtained (Table S1). Of them, the expression of 11,690 genes was detected ubiquitously in all seven wool follicle bulb samples from the three phases. In addition, we identified 12,964, 12,636 and 12,685 expressed genes in samples from the middle anagen, catagen and late telogen/early anagen phases, respectively. We chose the cut-off values of *p* ≤ 0.01 and expression level fold change ≥2 in the study. A total of 438 genes (136 upregulated and 302 downregulated genes, respectively) were identified to be differentially expressed in wool follicle bulb samples from the middle anagen phase compared to the catagen phase (Table S2). In wool follicle bulb samples from the catagen phase compared to the late telogen/early anagen phase, 203 differentially expressed genes (169 upregulated and 34 downregulated genes, respectively) were identified (Table S3). Additionally, 175 genes (30 upregulated and 145 downregulated genes, respectively) showed significant differences in expression level in wool follicle bulb samples from the late telogen/early anagen phase as compared to the samples from the middle anagen phase (Table S4). Nine differentially expressed genes with RPKM (reads per kilobase of exon per million fragments mapped) >5 were selected for validation of the sequencing results by quantitative real-time PCR (qPCR). The results showed a good agreement between qPCR and sequencing data (Figure 1). We also checked three marker genes by qPCR, *LEF1*, *TGFB1* and *TGFB2*, to confirm that our samples were collected from the right phases. Previous studies showed that the expression level of *LEF1* in hair follicles is higher in anagen compared with the catagen and telogen phases [15], whereas the expression level of *TGFB1* is upregulated in catagen [16], and the expression level of *TGFB2* is upregulated in late telogen/early anagen [17]. Both the qPCR results and sequencing data showed that the expression patterns of these three genes were consistent with the expected phases (Figure 2).

#### 2.1.2. Identified KRT and KRTAP Genes in Wool Follicle Bulb during the Cyclic Transformation

*KRT*s (keratins) and *KRTAP*s (keratin-associated proteins) are the most highly expressed gene families in wool follicle. Although only 52 *KRT* and 30 *KRTAP* genes were annotated in our data (Table S5), approximately 72% of the total RPKM values were from these *KRT* and *KRTAP* genes. Of the 82 *KRT* and *KRTAP* genes, only five *KRT* (*KRT5*, *KRT14*, *KRT17*, *KRT25* and *KRT27*) and two *KRTAP* genes (*KRTAP13-1* and *KRTAP9-2*) showed significant changes in expression during the wool follicle phase transformation (Table 1).

**Table 1:** Differentially expressed *KRT* and *KRTAP* genes in the wool follicle bulb of sheep during phase transformation.

Gene Symbol	Gene Expression (RPKM)			*p*-Value			Log2 Fold Change			Rank
	Middle Anagen	Catagen	Early Anagen	Middle Anagen/Catagen	Catagen/Early Anagen	Early Anagen/Middle Anagen	Middle Anagen/Catagen	Catagen/Early Anagen	Early Anagen/Middle Anagen	
**KRTs**										
KRT14	1191.45	2204.10	2489.06	0.018	0.846	0.010	−0.89	−0.18	1.06	35
KRT5	1017.81	1492.33	2120.69	0.104	0.340	0.007	−0.55	−0.51	1.06	39
KRT25	1137.49	1034.62	2451.84	0.880	0.012	0.007	0.14	−1.24	1.11	40
KRT17	653.00	1559.42	1697.52	0.005	0.931	0.004	−1.26	−0.12	1.38	46
KRT27	829.39	1149.58	1730.95	0.157	0.256	0.007	−0.47	−0.59	1.06	48
**KRTAPs**										
KRTAP13-1	21,205.73	10,454.46	17,439.53	0.006	0.098	0.311	1.02	−0.74	−0.28	9
KRTAP9-2	6320.34	3126.92	3490.35	0.010	0.859	0.016	1.02	−0.16	−0.86	21

#### 2.1.3. Gene Ontology and KEGG Pathway Analyses of the Differentially Expressed Genes in Wool Follicle Bulb during Middle Anagen-Catagen Transformation

**Figure 3:**
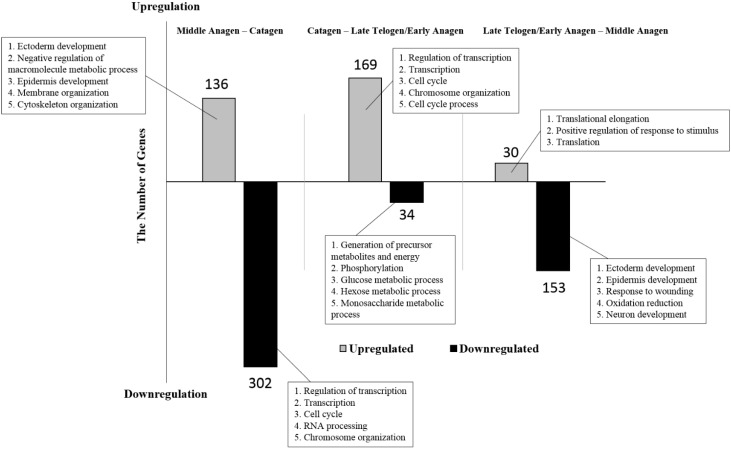
The differentially expressed genes during wool follicle phase transformation. A total of 438 genes (136 upregulated and 302 downregulated genes, respectively) were identified to be differentially expressed in wool follicle bulb samples from the middle anagen phase compared to the catagen phase. In wool follicle bulb samples from the catagen phase compared to the late telogen/early anagen phase, 203 differentially expressed genes (169 upregulated and 34 downregulated genes, respectively) were identified. Additionally, 175 genes (30 upregulated and 145 downregulated genes, respectively) showed significant differences in expression level in wool follicle bulb samples from the late telogen/early anagen phase as compared to the samples from the middle anagen phase. The boxes show the first five enriched GO terms for each group of differentially expressed genes.

Our study identified 438 differentially expressed genes in wool follicles of sheep between the middle anagen phase and the catagen phase, with 136 upregulated genes and 302 downregulated genes in the catagen phases (Table S2) (Figure 3). Gene Ontology analysis was performed, and we found that the most significantly enriched GO terms in the downregulated genes were involved in gene transcription and cell proliferation, including “regulation of transcription” (19.5%), “transcription” (17.2%), “cell cycle” (9.9%), “RNA processing” (9.6%) and “chromosome organization” (8.6%) (Table S6). The functional terms, which were related to the cellular catabolic process, were also highly enriched, such as “intracellular transport” (8.6%), “cellular macromolecule catabolic process” (8.6%), “macromolecule catabolic process” (8.6%), “proteolysis” (8.6%), and so on.

For the upregulated genes, the first five overrepresented functional terms included “ectoderm development” (7.4%),“negative regulation of macromolecule metabolic process” (7.4%), “epidermis development” (6.6%), “cytoskeleton organization” (5.9%), “regulation of peptidase activity” (5.1%), “growth” (4.4%), “regulation of cellular component size” (4.4%) and “negative regulation of molecular function” (4.4%) (Table S7). In addition, the specific enrichment terms for upregulated genes were related to negative regulation of molecular function and the macromolecule metabolic process. Notably, a group of upregulated genes in the catagen phase were found for the functional terms related to “negative regulation of macromolecule metabolic process” and “negative regulation of molecular function”, including *CST3*, *CSTB*, *S100A11*, *PPARD*, *CSTA*, *SIVA1*, *CCDC85B*, *FOXE1*, *VAX2*, *BAK1*, *ADRA1B*, *PKIA* and *HNF4A*.

Pathway analysis was performed, and 121 pathways were obtained with 106 differentially expressed genes between the middle anagen and catagen phases (Table S8). The pathway functions related to the cell proliferation, differentiation and apoptosis were obtained, such as the ErbB, MAPK and apoptosis signaling pathways. Some well-known pathways related to the regulation of wool follicle development cycle were also found, including TGF-beta/BMP and Wnt signaling pathways.

#### 2.1.4. Gene Ontology and KEGG Pathway Analyses of Differentially Expressed Genes in the Wool Follicle Bulb during Catagen-Late Telogen/Early Anagen Transformation

The comparison of data from catagen and late telogen/early anagen revealed 203 differentially expressed genes with 169 upregulated and 34 downregulated genes (Table S3) (Figure 3). For the upregulated genes, the strongest overrepresentation functional term was “regulation of transcription” (20.7%). Further overrepresented functional terms included, for example, “cell cycle” (10.1%), “chromosome organization” (9.5%), “DNA metabolic process” (8.9%), “cellular response to stress” (8.9%), “cell adhesion” (8.3%), “cell division” (7.7%), “RNA processing” (7.7%), and so on (Table S9). The number of the downregulated genes was much less than that of the upregulated genes; thus, fewer overrepresented functional terms were found, such as “glucose metabolic process” (8.8%) and “negative regulation of apoptosis” (8.8%) (Table S10). The comparison of downregulated and upregulated genes revealed that the functional terms related to positive regulation of cellular biosynthetic process and cell migration were specifically enriched for the upregulated genes in early anagen, as well as the terms related to negative regulation of apoptosis were specifically enriched for the downregulated genes.

The results of pathway analysis revealed 86 pathways with 60 differently expressed genes (Table S11). The first five overrepresented pathways were “focal adhesion”, “vascular smooth muscle contraction”, “leukocyte transendothelial migration”, “spliceosome”, “tight junction” and “regulation of actin cytoskeleton”.

#### 2.1.5. Gene Ontology Analyses of Differentially Expressed Genes in the Wool Follicle Bulb during Late Telogen/Early Anagen-Middle Anagen Development

We also compared the data from late telogen/early anagen and middle anagen, resulting in 183 differentially expressed genes with 30 upregulated and 153 downregulated genes (Table S4) (Figure 3). For the upregulated genes, only three functional terms were found, including “translational elongation” (10%), “positive regulation of response to stimulus” (10%) and “translation” (10%) (Table S12). The functional terms for the downregulated were related to epidermis development and hair cycle, such as “epidermis development” (9.2%), “cell proliferation” (5.2%), “cell motion” (5.2%), “regulation of protein kinase activity” (4.6%), “epidermal cell differentiation” (3.3%) and “hair cycle process” (2.6%) (Table S13).

### 2.2. Discussion

Cells in the hair bulb of the follicle proliferate rapidly to generate the hair shaft. During the growth cycle of hair, the hair follicle bulb undergoes regression and regeneration in response to the factors from both the macroenvironment (such as skin and adipose) and microenvironment (such as dermal papilla and hair germ in bulb) [18]. Previous studies were mainly focused on the investigation of the regulators in the macroenvironment. Due to the fact that the intrinsic factors in the bulb also play important roles in controlling the hair follicle cycling and the wool fiber properties, the aim of the present study was to determine the gene expression patterns in hair follicle bulb during the regression (middle anagen-catagen transformation) and regeneration (catagen-late telogen/early anagen transformation) phases in sheep, and the results revealed that groups of genes might act as the wool follicle bulb and wool fiber growth-related activators/inhibitors in sheep.

**Figure 4:**
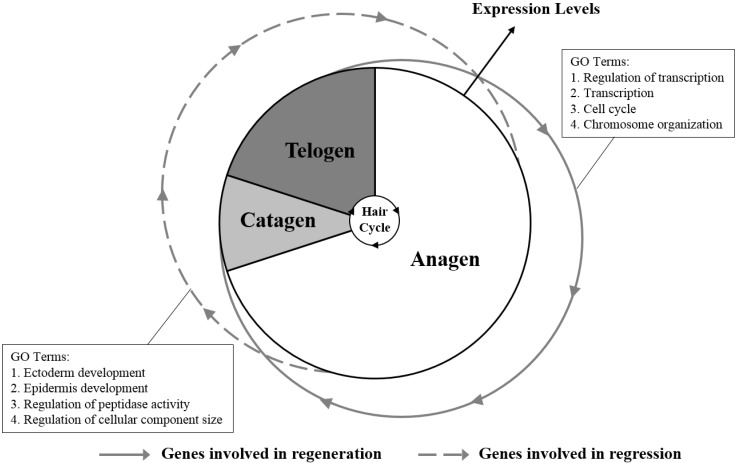
The gene expression patterns during the wool follicle bulb regression and regeneration. Two groups of genes that presented the distinct expression patterns during the phase-transformation may have important roles for the wool follicle bulb regression and regeneration. Of these two groups of genes, one group of them was upregulated in catagen, then downregulated in the middle anagen of the next cycle (regression-associated genes). The GO terms analysis revealed that these genes were related to epidermis development, cell morphology and apoptosis. Whereas the other group of genes were upregulated in the late telogen/early anagen phase, then downregulated in the catagen phase (regeneration-associated genes), these genes were related to cell proliferation and growth.

Although the structural patterns of the hairs are similar among closely-related mammalian taxa, the patterns of hair follicle cycling differ in different species. Unlike the mosaic pattern of hair follicle cycling in humans and the wave pattern of hair follicle cycling in mouse, the wool follicle cycling presents the seasonal pattern in sheep [19]. It has been demonstrated that the cycling of the hair follicle is regulated by a complex signaling network, including activators and inhibitors in human and mouse [20], and the regression and regeneration of the hair follicle will be achieved based on the balanced activities of the inhibitors and activators [18,21]. Our data presented the expression patterns of the genes in the wool follicle bulb during the wool follicle cycling in sheep. We also found two groups of genes that exhibited opposite expression patterns during the periodic wool follicle bulb regression and regeneration (Figure 4). The genes in one group (termed the regression-associated genes) were upregulated in catagen and gradually downregulated in late telogen/early anagen and middle anagen of the next cycle (Table S14). The GO terms analysis revealed that these genes were related to epidermis development, cell morphology and apoptosis, which may contribute to inducing the primary features of the hair follicle in the catagen phase, including cessation of HMC proliferation and differentiation, formation of the club structure and recession of the epithelial strand [2]. Whereas the genes in the other group (termed the regeneration-associated) were upregulated in the late telogen/early anagen phase and downregulated in the wool follicle bulb during middle anagen-catagen phase transformation (Table S15), these genes were found to be related to cell proliferation and growth. Thus, these data revealed that two groups of genes might play roles in the modulation of the wool follicle bulb regression and regeneration during the follicle cycling and could be considered as candidate genes for further investigation.

The catagen phase is the regressive phase with an extensive apoptosis process in the HF [22]. Of the genes that were upregulated in the catagen phase, *VAX2* has been shown to be one of the inhibitors of the *Wnt* signaling pathway. It could regulate the expression of a truncated TCF7L2 isoform, which acts as a dominant-negative *Wnt* antagonist [23]. *Wnt* signaling is one of the important pathways that contributes to promoting wool follicle growth. Thus, our finding that *VAX2* was upregulated in the catagen phase suggests that *VAX2* might participate in the regulation of wool follicle bulb regression via inhibiting the *Wnt* signaling. Additionally, not only the genes having a pro-apoptotic function, but also those having an anti-apoptotic role were found to be upregulated in the catagen phase. For example, *CCDC85B* and *PKIA* have been found to be the repressors of gene transcription [24,25], as well as *SIVA1* and *BAK1* genes determined as the inducers of apoptosis [26,27]. However, *CSTA*, *CSTB* and *CST3* are members of the *CST* family and have been suggested to have critical roles for the inhibition of the function of the lysosomal proteinases and cysteine proteases [28]. In addition, *S100A11* has been reported to affect the maintenance of p21-CIP1 protein stability and inhibits apoptosis in keratinocytes [29]. The hair follicle is a well-organized multicellular structure. During the apoptosis-driven involution phase, although certain cells, such as the matrix cell, undergo apoptosis, some hair bulb cells can survive [30]. Therefore, the balance between the pro- and anti-apoptotic regulatory components is required for the maintenance of the HF structure and the renewal of further regeneration.

KRTs (keratins) and KRTAPs (keratin-associated proteins) are the primary structural proteins of wool fiber. According to the new nomenclature system, the keratins are mainly divided into three categories: (1) epithelial keratins; (2) hair follicle-specific epithelial keratins; and (3) hair keratins [31]. In humans, a total of 54 functional keratin genes have been identified to date. In this study, the expression of 52 keratin genes was detected in the wool follicle, and of them, 47 keratin genes, including all 15 hair keratin genes detected, did not show significant changes in expression levels during the wool follicle cycling. This result is expected as the function of the KRTs in structural support to the epithelial cells has been demonstrated. The other five keratin genes (*KRT5*, *KRT14*, *KRT17*, *KRT25* and *KRT27*) were identified to be expressed differentially during the phase transformation. *KRT17*, which belongs to the category of epithelial keratin genes, was detected to be expressed in the hair matrix and the early differentiating hair epithelial cells in the lower portions of the hair shaft, as well as the outer root sheath in adult mouse skin [31,32]. During the anagen phase, the matrix epithelial cells within the hair bulb proliferate rapidly. However, the proliferation of the matrix epithelial cells ceases and apoptosis occurs in the catagen phase. Studies suggested that KRT17 was involved in the regulation of hair follicle cycling. For example, KRT17 was found to be required for the maintenance of the anagen state in hair follicles [33,34], and it also could play a positive role in the regulation of cellular adhesion [35]. In our data, we found that the expression of *KRT17* was significantly raised in catagen and early anagen phases, but downregulated in the middle anagen phase, indicating that KRT17 might be a positive regulator for the anagen phase (wool follicle growth) and a negative antagonist in the catagen phase (wool follicle regression). Previous research showed that the two epithelial keratins, KRT5 and KRT14, function in the modulation of epithelial cell proliferation and differentiation via the *PI3K/Akt* and *Notch1* signaling pathways, respectively [36]. In our data, we found that the expression of *KRT5* and *KRT14* was significantly increased in early anagen, suggesting that *KRT5* and *KRT14* might play a role in the regulation of the formation of wool follicle structure in early anagen. We also found that *KRT25* and *KRT27*, which belong to the category of hair follicle-specific epithelial keratin genes, were decreased in the middle anagen phase, but significantly raised their expression in the catagen and early anagen phases. Previous studies revealed that *KRT25* and *KRT27* were located at the IRS of the hair follicle [37]. Thus, we predict that these two *KRT* genes might be related to the formation of IRS layers of wool follicle in the early anagen phase. The keratin-associated proteins (KRTAPs) function in the formation of the rigid hair shaft through a cross-link with the keratin intermediate filament proteins via extensive disulfide bonding [4]. A total of 31 *KRTAP* genes were identified to be expressed in the sheep wool follicles in this study, and two of them, *KRTAP13-1* and *KRTAP9-2*, were significantly differentially expressed during the three phases. The fiber and wool properties are dependent on the composition and interactions of KRTs and KRTAPs. Although, most of the *KRT* and *KRTAP* genes are evolutionarily conserved, the expression patterns of the two types of genes in wool follicles have some differences compared to the human or mouse orthologs, due to the distinctive features of hair and wool [9]. Thus, our data could be useful in finding genes controlling the hair shaft keratinization in sheep.

## 3. Experimental Section

### 3.1. Ethics Statement

All research involving animals was conducted according to Regulation No. 5 of the Standing Committee of Hubei People’s Congress and was approved by the Standing Committee of Hubei People’s Congress and the ethics committee of Huazhong Agricultural University, Wuhan, China. The ethics committee of Huazhong Agricultural University, China, approved this study, and the approved permit number for this study is “HBAC20091138”.

### 3.2. Animal and Sample Collection

In this study, three female 2-year-old Tibetan sheep (*Ovis aries*) were from the Agriculture and Animal Husbandry College of Tibet in Tibet, China. The sheep were fed for one year, and 3 time points were selected to collect wool follicle bulb samples, including the middle anagen phase (May, 2011), the catagen phase (October, 2011) and the late telogen/early anagen phase (January, 2012). For each time point, the wool follicle bulb samples were collected from the lateral body of the 3 sheep, and the biological replicate samples were not pooled together. In total, 9 wool follicle bulb samples (3 phases × 3 sheep) were collected during the 3 follicle cyclic phases. Each of the wool follicle bulb samples was homogenized completely in TRIzol reagent (Invitrogen, Carlsbad, CA, USA) immediately after collection and then centrifuged at 12,000 rpm and 4 °C for 10 min. The cleared supernatant was transferred to a fresh tube and stored at −80 °C.

### 3.3. cDNA Library Construction and Illumina Sequencing

Total RNA was isolated according to the instructions of the TRIzol reagent (Invitrogen) and purified by the mRNA purification kit (Promega, Madison, WI, USA) to enrich mRNA. For the first-strand cDNA synthesis, mRNAs were reverse-transcribed by Powerscript II (Takara, Dalian, Liaoning, China) with PCR primers SMART IV Oligonucleotide and CDS III/3' PCR Primer (See Clontech SMART cDNA Library Construction Kit User Manual, Mountain View, CA, USA). Double-strand cDNA amplification was performed with LA Taq enzyme (Takara, Dalian, Liaoning, China) for 25 cycles (95 °C for 30 s, 68 °C for 8 min). Finally, double-strand cDNA was purified using the DNA purification kit (Qiagen, Hilden, Germany). Approximately 10 μg of cDNA were used for Illumina sequencing library construction according to the manufacturer’s protocols. Libraries were prepared from a 300–500-bp size-selected fraction following adapter ligation and agarose gel separation. In total, 7 libraries were constructed (3 for the middle anagen phase, 2 for the catagen phase and 2 for the late telogen/early anagen phase, respectively), because 2 of the 9 RNA samples failed to meet the quality criteria for library construction. The constructed libraries were sequenced using a single end read protocol with 75 bp of data collected per run on the Illumina Genome Analyzer (Illumina, San Diego, CA, USA). Data analysis and base calling were performed by the Illumina instrument software (Illumina, San Diego, CA, USA).

### 3.4. Quantitative Real-Time RT-PCR

The same total RNA samples for Illumina sequencing were used for qPCR validation and reverse-transcribed by PrimeScript RT Reagent Kit with gDNA Eraser (Takara, Dalian, Liaoning, China). The qPCR assay was performed by SYBR Green All-in-One QPCR Mix (Genecopoeia, Rockville, MD, USA). Approximately 5 ng of cDNA were used for each qPCR reaction. The sheep GAPDH gene was used as the reference gene. An annealing temperature of 60 °C was used for all the genes. The 2^−∆∆*C*t^ method was used to analyze the expression level [38].

### 3.5. Annotation and Differentially Expressed Gene Identification

The mapping work was performed by Bowtie software with default parameters (http://bowtie-bio.sourceforge.net/index.shtml) [39]. The gene annotation and expression level normalization was performed by the script of the HTSeq software (http://www-huber.embl.de/users/anders/HTSeq/doc/overview.html). Due to the incomplete information for the sheep reference, the sequence information of the genes from cattle, which are highly evolutionarily homologous with sheep, was used. The database was UMD3.1 (Center for Bioinformatics and Computational Biology, University of Maryland, Bos taurus assembly Database version: 3.1) (ftp://ftp.ncbi.nlm.nih.gov/genomes/Bos_taurus/). The differentially expressed genes were analyzed by edgeR software (http://www.bioconductor.org/packages/release/bioc/html/edgeR.html). All of the analyses were performed using default parameters.

### 3.6. Gene Functional Annotation

The Gene Ontology (GO) and pathways analyses were performed by the “Functional Annotation” tools from the DAVID 6.7 website (http://david.abcc.ncifcrf.gov/) [40]. The *Homo sapiens* species database was selected for all of the analyses. For GO analysis, default parameters were used, and the results of the “GOTERM_BP_FAT” class are presented in this study. For pathway analysis, the KEGG pathway database was selected. The parameters were as follow: count = 2 and EASE = 1.0. “Count” means the threshold of minimum gene counts belonging to an annotation term, and “EASE” is a modified Fisher exact *p*-value.

## 4. Conclusions

In conclusion, this study has investigated the gene expression profiles of the hair bulb during the follicle regression (middle anagen-catagen transformation) and regeneration (catagen-late telogen/early anagen transformation) in sheep. Two groups of genes that present the distinct expression patterns during the phase-transformation may have important roles for the wool follicle bulb regression and regeneration. In addition, the *KRT* and *KRTAP* genes identified in the study are potential candidate genes in the regulation of the formation and keratinization of wool shaft in sheep. Our results presented in the study add new data towards an understanding of the mechanisms involved in wool fiber growth in sheep.

## Supplementary Materials

Supplementary materials can be found at http://www.mdpi.com/1422-0067/16/05/9152/s1.
